# Spatial prediction of the probability of liver fluke infection using a geographic weighted regression (GWR) model in waterways connecting the Mekong River, Sakon Nakhon of Thailand

**DOI:** 10.1016/j.onehlt.2026.101320

**Published:** 2026-01-05

**Authors:** Benjamabhorn Pumhirunroj, Patiwat Littidej, Thidarut Boonmars, Atchara Artchayasawat, Nutchanat Buasri, Donald Slack

**Affiliations:** a Program in Animal Science, Faculty of Agricultural Technology, Sakon Nakhon Rajabhat University, Sakon Nakhon 47000, Thailand; b Research Unit of Geoinformatics for Spatial Management, Department of Geoinformatics, Faculty of Informatics, Mahasarakham University, Maha Sarakham 44150, Thailand; c Department of Parasitology, Faculty of Medicine, Khon Kaen University, Khon Kaen 40002, Thailand; d Department of Agriculture and Resources, Faculty of Natural Resources and Agro-Industry, Kasetsart University, Chalermphrakiat Sakon Nakhon Province Campus, Sakon Nakhon 47000, Thailand; e Department of Civil & Architectural Engineering & Mechanics, University of Arizona, 1209 E. Second St., P.O. Box 210072, Tucson, AZ 85721, USA

**Keywords:** Spatial prediction, Liver fluke infection, Optimized geo-weighted regression (GWR), Hexagonal grid, Mekong River, Sakon Nakhon of Thailand

## Abstract

**Introduction:**

Liver flukes (*Opisthorchis viverrine, OV*) infections in water sources continue to persist in Sakon Nakhon Province, which is linked to the Mekong River. The agency's traditional infection data comprises the locations of infected water sources. However, this data is insufficient for developing a predictive model for infections within the sub-basin. When analyzed alongside independent variables, represented as identical points, it lacks the necessary information to generate a trend line that produces a reliable coefficient. This study implemented a spatial model that integrates a geographic-weighted regression (GWR) framework with appropriate weighting as a prototype. This approach improves the selection of independent variables by shifting from a point-based methodology to a weighted hexagonal grid.

**Method:**

A set of eight independent variables land use, soil drainage, road network, water sources, streamlines, surface temperature, NDMI (Normalized Difference Moisture Index), and NDVI (Normalized Difference Vegetation Index) was initially weighted. This study developed three linear models based on the Geographically Weighted Regression (GWR) model. It demonstrates the advantages of utilizing a hexagonal grid instead of a point grid. The three alternative models were tested with various independent variables and employed a factor-by-factor averaging approach, which necessitates the hexagonal grid size as a counterweight to ensure fairness across the entire grid, rather than relying solely on point data. A mathematical model was developed to calculate the average of each factor in order to achieve equality across a hexagonal grid area. Subsequently, the correlation was tested, and the alternative models were grouped. The resulting dendrogram includes three models.

**Results and discussion:**

The results of the GWR comparison test were derived from both infected and hexagonal water source data. Models constructed from hexagonal grids consistently outperformed all alternative models, with R^2^ values improving to 58.7 %, 41.1 %, and 53.2 % for Model-1, Model-2, and Model-3, respectively. The RMSE also showed significant improvement, decreasing to 77.1 %, 60.2 %, and 67.2 %. Additionally, the model's accuracy was evaluated using AUC values of 0.725, 0.652, and 0.707, indicating that the developed model can effectively predict water source infections. Model-1 emerged as the most representative across all tests, incorporating soil drainage factors and road proximity as key influences on water source infection. Finally, the results are presented as infection prediction maps for each grid, highlighting areas of both overestimation and underestimation. The most accurate prediction model identified that over 95 % of grids had a high degree of accuracy. This study is anticipated to be applicable to infections caused by other water-mediated parasites.

## 1 Introduction

**Fig. 1:**
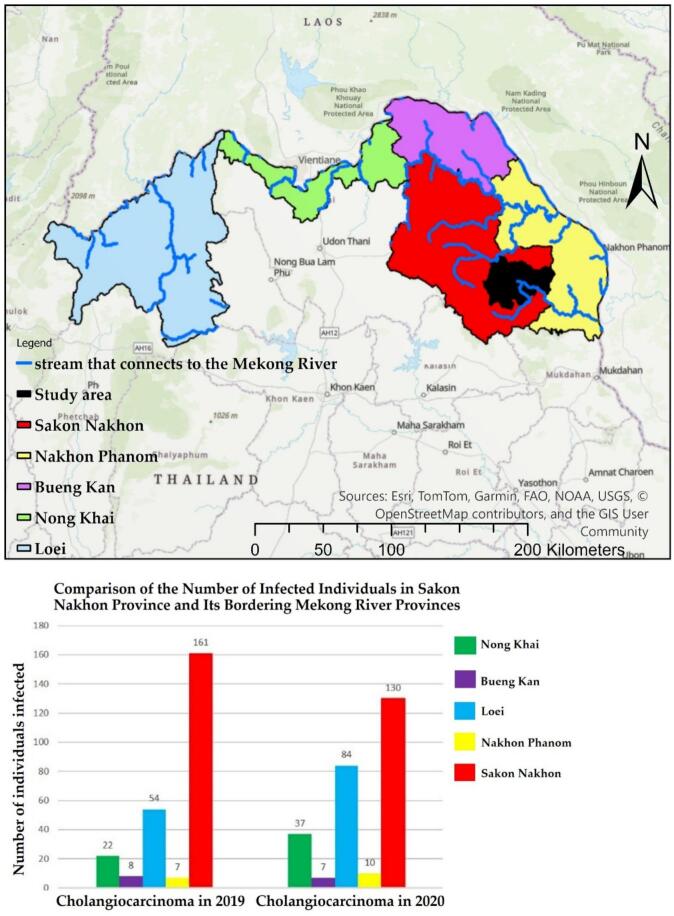
Statistics of provinces with high CCA and OV infections, along with comparative data from the Sakon Nakhon Province study area, applied from [1].

Severe liver fluke infections have been reported in the provinces bordering the Mekong River in northeastern Thailand. However, historical statistics indicate that Sakon Nakhon Province, which is not adjacent to the Mekong River, has a higher number of cases than the aforementioned provinces [1]. The hepatic leafworm, Opisthorchis viverrine (OV), is associated with the development of cholangiocarcinoma (CCA), which is a form of bile duct cancer. [[2], [3], [4]]. Thailand exhibits the highest prevalence of cancer cases [5]. Infections caused by this leafworm have also been linked to the consumption of fermented fish products [6]. Each year, Sakon Nakhon Hospital identifies nearly a thousand new cases of CCA. Despite the established major risk factors associated with OV infection, the incidence of CCA has not decreased in recent decades [7,8]. Statistical data from a study by [1] indicates that provinces with high infection rates are typically located near the Mekong River, including Loei, Nong Khai, Bueng Kan, and Nakhon Phanom. Interestingly, the study reveals that Sakon Nakhon Province has a higher number of infections than all the aforementioned provinces [[9], [10], [11]]. This raises questions about the reasons for the elevated infection levels in Sakon Nakhon, as illustrated in the spatial relationship shown in Fig. 1.

Individuals with a high severity of OV infection (exceeding 6000 eggs/g of feces) are 14.1 times more likely to develop CCA compared to those who are not infected [1]. About 10 % of individuals infected with OV develop CCA, resulting in significant health crises in the region [12,13]. The five-year survival rates for patients undergoing surgery for intrahepatic, distal extrahepatic, and hilar CCA are 22–44 %, 27–37 %, and 11–41 %, respectively, according to [14].

The confluence of the Mekong River and Nong Harn Lake in Sakon Nakhon Province is characterized by a densely populated area that contains numerous gastric juice flow lines throughout the basin. This environment offers an optimal habitat for intermediate hosts and supports a robust fish population, which is essential for the local community's sustenance. Fish serves as the primary source of protein for residents in the river basin. It is commonly enjoyed both raw and cooked with herbs, resulting in a sweet, sour, and spicy flavor [4,15]. For this reason, residents living near river basins frequently incorporate fish into their daily meals. Preliminary screening results from 2019 to 2021 indicate that only a small percentage of individuals are infected with hepatic leafworm parasites [2]. Research on the prevalence of helminthiasis infection in cod liver (contagious larvae) [12] revealed that a study conducted between 2016 and 2017 found a density of 10 to 20 metacercaria per kilogram of fish [1]. Sakon Nakhon province is currently experiencing an outbreak of hepatitis leafworm disease. This is largely due to feces containing parasite eggs contaminating water sources, resulting in recurrent illnesses and a cycle of infection. The primary factor contributing to this ongoing cycle is the use of manure fertilizer from vegetable plots during the dry season. To address this issue, spatial analysis using geographic information systems (GIS) and remote sensing (RS) is essential for understanding the topographical complexities of the interconnected small watersheds.

The application of GIS knowledge as an analytical tool is highly valuable in the study of hepatic helminthiasis infections. Utilizing remote sensing information derived from satellite imagery enables comprehensive spatial analysis of the probability and distribution of liver flukes, as well as the preparation of temporal data reflecting changes in surface humidity. This analysis may incorporate indicators such as the Standard Vegetation Index, Soil Moisture Index, Soil Cover Index, and other relevant indices associated with the presence of liver fluke intermediates [13,16].

Many studies have used spatial statistics to examine the relationship between geographical factors and liver fluke infection [17]. However, some studies have reported conflicting results and inconsistencies in the raster data, often due to the analysis of large areas [18,19]. Studies aimed at predicting the prevalence of infection over large areas can effectively utilize general spatial linear models, such as Ordinary Least Squares (OLS). However, when applied to small watersheds, these models exhibit limited efficiency, likely due to challenges in accurately representing independent variables. As a result, models that can be segmented into spatial units to more accurately reflect the actual values of independent variables offer a viable alternative for studying spatial infection prediction in small watershed systems. Conversely, other research [1,[20], [21], [22], [23], [24]] has focused on developing geographically weighted regression (GWR) models for smaller areas to analyze hydrological factors, yielding high R^2^ values across all models. A study by [25] demonstrated that the combination of appropriate spatial modeling with mathematical models can enhance the accuracy of linear models. The principles of geo-statistics [26], particularly the GWR modeling method, necessitate the creation of sub-spatial units [27], such as sub-basins. These sub-basins are defined between the flow boundary and the modeling control boundary, and they are essential for accurately analyzing the various indices that need to be constructed as independent variables. As noted by [28], this requirement enhances the effectiveness of GWR models in forecasting and understanding spatial correlations. In a study [20,21], it was found that the creation of hexagonal spatial units can produce data sets that enhance the geographic weight of the Geographically Weighted Regression (GWR) model more effectively than traditional spatial units, such as points or polygons.

To develop spatial models for analyzing relationships in small areas, such as sub-basins [1,20,24], it is crucial to select appropriate models and adjust the sub-area units according to the data distribution and the dependent and independent variables. Relying exclusively on OLS models in independent multivariate analysis often results in low accuracy due to the many independent factors that contribute to the variability of the model.

In this study, GWR modeling was employed to analyze the relationship between various independent variables and the proportion of infections prior to OV. Unlike previous spatial modeling research, which did not utilize GWR models or consider sub-spatial unit boundaries in small watershed systems to detect liver fluke infections, this study seeks to identify the independent variables associated with spatial infections. Additionally, it aims to accurately model these variables using a limited set of interconnected independent variables through GWR modeling. This involves a comparison of the performance of the model derived from the traditional method in the standard area unit with that of the model developed using weighting in the hexagonal area unit. Effective management at the sub-basin level can ensure protection, provided that the spatial distribution of each parasite's features is significant within each sub-basin unit [29]. For instance, by disrupting the mollusc host cycle, we can enhance population wellbeing and prevent future diseases, leading to reduced community impact and lower medical expenses. The study established guidelines for using the model developed to investigate the spatial characteristics of liver fluke infection. The primary challenge encountered due to the limited quantity of variable data and its distribution, which does not encompass the entire study area, is that the model fails to yield a reliable decision coefficient. Traditional modeling approaches have demonstrated that using point data as representatives is inadequate for accurately reflecting the watershed, as multiple points may be necessary. In contrast, employing a hexagonal grid allows for the averaging of multiple points within each grid cell, thereby reducing the variability of maximum or minimum values. Consequently, this study is significant for advancing spatial models aimed at analyzing OV infections in the subbasin. It develops data on hexagonal grids that enhance the predictive capabilities of independent variables, resulting in models that outperform traditional point-based approaches.

The primary objectives are to analyze the spatial factors linked to human liver fluke infection using hexagonal boundaries and to optimize an alternative model of GWR. This approach aims to improve public health management strategies to reduce the risk of liver fluke infection in humans.

## 2 Materials and methods

### 2.1 The study area

**Fig. 2:**
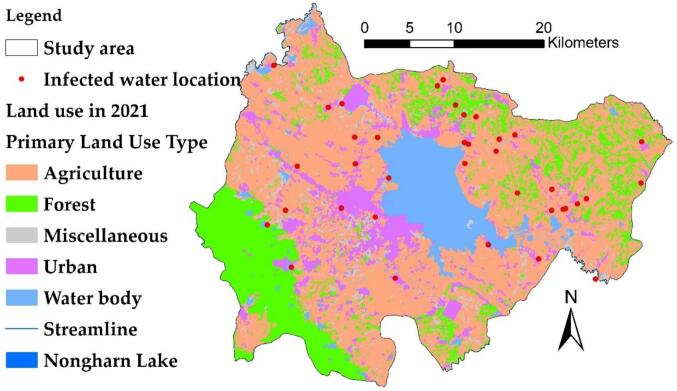
Infection locations and land use.

Phonna Kaew District and Mueang Sakon Nakhon District are located in Sakon Nakhon Province in Northeast Thailand. Phonna Kaew District is approximately at 17.2167° N latitude and 104.1667° E longitude, situated about 30 km east of Sakon Nakhon City. Mueang Sakon Nakhon District, the administrative center of the province, is found at approximately 17.1545° N latitude and 104.1456° E longitude, in the central region of Sakon Nakhon. Both districts are abundant in freshwater resources, especially the Nong Harn Lake, which is a vital water source that significantly impacts the local ecosystem and the distribution of liver fluke in the area, as shown in Fig. 2. The majority of contaminated water sources are located in rice farming regions, which comprise 92 % of the total area. The average size of the rice fields in adjacent plots is approximately 327 acres. Additionally, land use data from 2019 to 2021 indicates that there has been little change in land utilization within the affected area. This stability can be attributed to the farmers' reliance on water from Nong Han, which is available year-round, leading to infrequent transitions to other types of agriculture. These two districts are situated east of the Songkhram River Basin, near Nakhon Phanom Province. Nong Harn Lake is located approximately 40 km from the Mekong River, facilitating the exchange of fish between the two bodies of water. Phonna Kaew District serves as a junction for the water source and the main river, connecting through the Songkhram River. This connection allows for the movement of fish from the Mekong River and its tributaries, potentially increasing the risk of liver leafworm infections in the fish.

In the northeast of Thailand, there are long-standing public health problems of liver fluke and cholangiocarcinoma. It kills at least 20,000 people in the Northeast annually [30,31]. Therefore, detecting this infection is of great importance. Getting rid of parasites can reduce the risk of developing bile duct cancer [34]. The data on liver fluke infections used in this study will be generated as variable data based on the percentage of infected people obtained from the Sakon Nakhon Provincial Public Health Office (SKKO) [1] (https://skko.moph.go.th/dward/web/index.php?module=skkoMOHPA). The long-standing screening technique is the modified Kato-Katz method, which has proven to be effective in the past during widespread parasite outbreaks. It can be used to carefully examine parasite eggs in the feces. The results of fecal analysis conducted to detect eggs of *O. viverrini* immediately after sample collection from [32] indicated that the majority of infected individuals were located in the Phonna Kaew and Mueang Sakon Nakhon districts. The infection rate was found to increase among those aged 18 to 80 years. The office has suggested an alternative method that offers greater accuracy than the stool test, although it comes with a relatively high budget. This method includes the enzyme-linked immunosorbent assay (ELISA) and the formalin-ethyl acetate concentration technique (FECT) [29,32,33]. However, the traditional methods currently employed remain reliable for testing large sample sizes, as seen in this study.

**Fig. 3:**
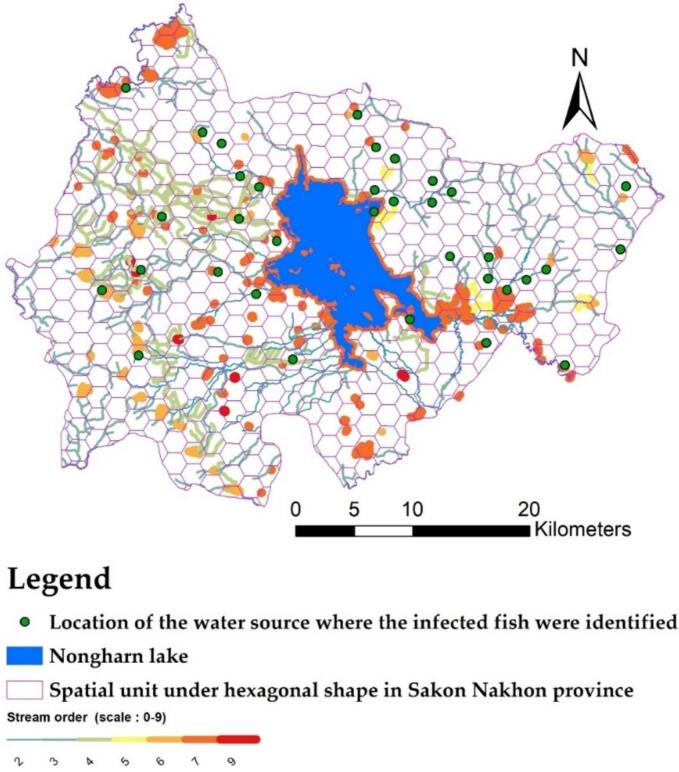
The study area and the distribution of the prevalence of ov-infection, presented in percentage (point format) [1].

According to the statistics, the highest number of cases was recorded in the Phonna Kaew and Mueang Sakon Nakhon districts. Fig. 3 illustrates the locations of infected individuals by village, along with the sub-river that connects upstream to downstream levels (0–9). Between 2019 and 2020, a total of 12,063 stool samples were tested nationwide, and the results were reported to Public Health District 8 (District 8) (Office, 2021) [23]. Of these, 2832 samples originated from Sakon Nakhon province. The high number of infections is often associated with river basins that can flow together during the dry season [34]. The authorities are primarily concerned with the risk of infection. Consequently, accurately predicting the percentage of infected individuals is essential for effectively managing liver fluke infections within a spatial context.

### 2.2 Optimal design of hexagonal grid size

**Fig. 4:**
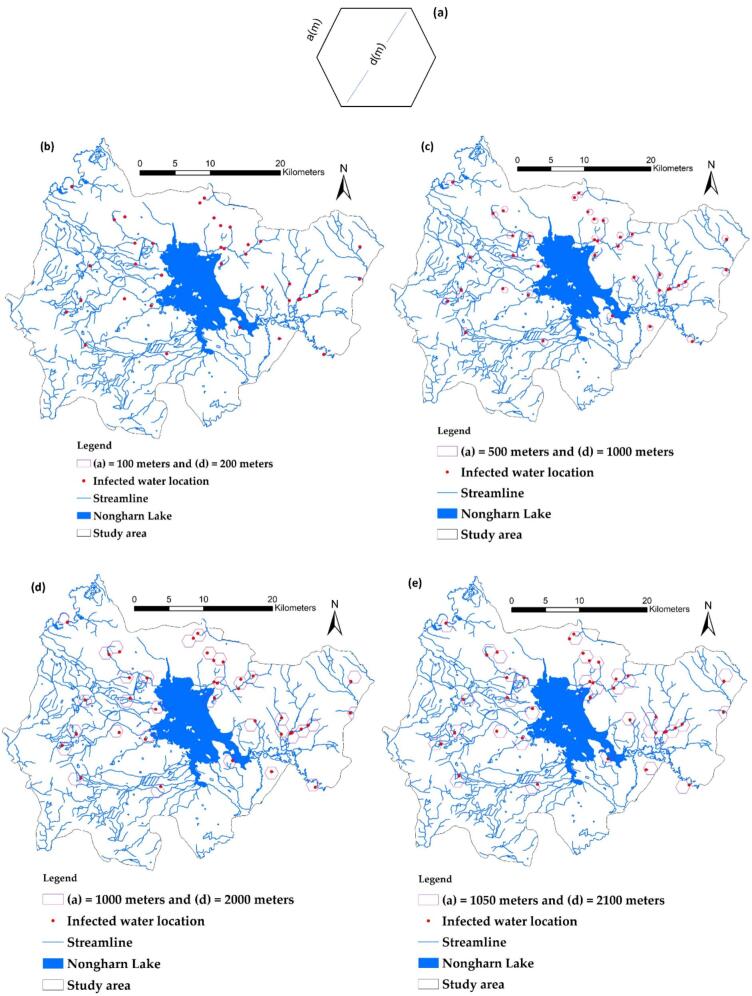

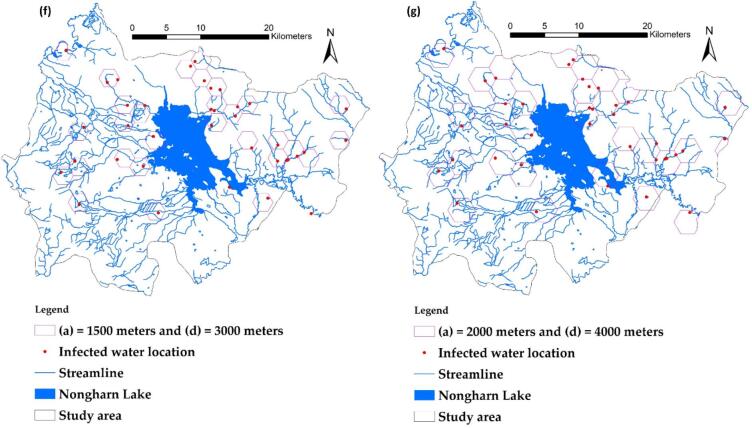
Variable elements of the hexagonal grid used for calculations (a), The relationship between the diagonal length (d) of the hexagonal grid (in meters) and H_d_ is illustrated in Fig. 4(b) through 4(h).

In this study, an index for determining the dimensions of the hexagonal grid was developed using a method known as the H_d_ index. Determining the appropriate size of the grid diagonal will create a grid configuration that addresses a wide range of factors and reduces data variance caused by excessive jump values. It is crucial to identify the side length (a) and diagonal (d) to assess the appropriate H_d_ value for modeling, as shown in Fig. 4(a). The variables of the hexagonal grid are presented, followed by images 4(b) through 4(g), which illustrate distance designations of 100 m, 500 m, 1000 m, 1050 m, 1500 m, and up to 2000 m, respectively. This analysis aims to observe the trend of H_d_ to determine if any further changes are evident.

**Fig. 5:**
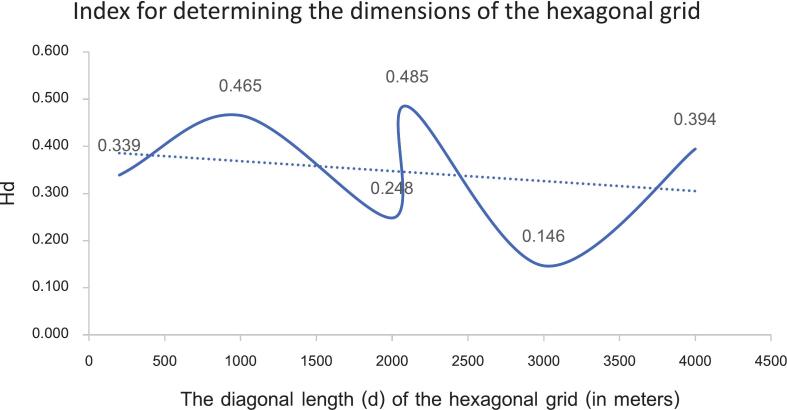
Relationship between the diagonal length (d) of the hexagonal grid (in meters) and H_d_.

Experts from local agencies have assigned a 35 % importance to the size of the hexagonal grid (A_K_). A higher value indicates a more effective model for capturing the values of multiple independent variable factors. Following this, the number of grids that do not cover the waterline (NNS) is prioritized at 30 %. Conversely, the number of grids that do cover the streamline (N_s_) is also taken into account. Lastly, the criterion deemed least important is the number of infection points within the grid (N_i_). Each factor will be normalized to a measurable range of 0 to 1 before being used in the calculation for H_d_, as shown in Table 1. It was determined that the diagonal length corresponding to the highest H_d_ (0.485) value was d = 2100 m, as illustrated in Fig. 5. This measurement was utilized to extract the values of the dependent and independent variables within this grid size. The grid created for training and testing points is made up of full hexagon sizes. Areas next to district or provincial boundaries that do not form complete hexagons will not be included in the calculation of the H_d_ index. The data models of various independent variables, including both vector and raster formats, are combined into new attributes in vector form to enable faster processing with the GWR model. The configuration of the grid size varies based on the study area, owing to the differing numbers and distributions of infection sites in each location; however, this diagonal spacing approach remains applicable.

**Table 1:** Data extracted from each d size, along with standard adjustments, is utilized to calculate H_d_.

a(m)	d(m)	Area(m^2^), (A_k_)	Number of grids obtained (N)		Number of infected points within the grid (Ni)		Number of grids covering streamlines (Ns)		Number of grids non-covering streamlines (Nns)			H_d_
		Normalized (maximized)				Normalized (maximized)		Normalized (maximized)		Normalized (minimized)		(A_k_)*0.35 + (N_i_)*0.1 + (N_s_)*0.25 + (Nn_s_)*0.3
100	200	25,981	0.000	60,653	1.000	1	0.000	37	0.556	2	0.667	0.339
500	1000	649,519	0.060	2560	0.039	2	0.333	36	0.444	1	1.000	0.465
1000	2000	2,598,076	0.248	682	0.008	2	0.333	33	0.111	3	0.333	0.248
1050	2100	2,864,379	0.274	615	0.007	2	0.333	34	0.222	1	1.000	0.485
1500	3000	5,845,671	0.561	322	0.002	2	0.333	29	−0.333	4	0.000	0.146
2000	4000	10,392,305	1.000	188	0.000	4	1.000	30	−0.222	4	0.000	0.394

### 2.3 Optimized spatial weighting of independent variables on a hexagonal grid

**Fig. 6:**
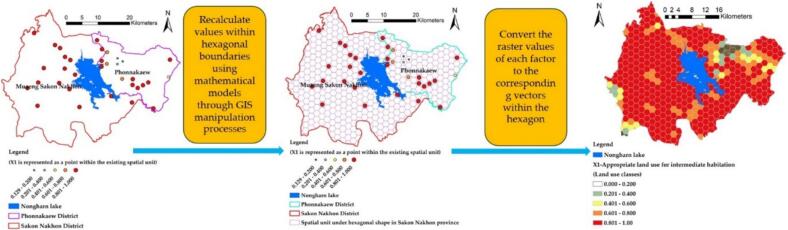
Optimized spatial weighting method for independent variables on a hexagonal grid.

The development of a spatial model to predict the percentage of infected individuals relies on variable data (Y) from previous studies. Most of these studies focus on generating point data to represent the locations of infected water sources, which facilitates the storage of spatial data. However, using vector data does not adequately capture the relationships among the various independent variables that influence the dependent variable. This limitation necessitates the inclusion of a large set of independent variables to identify the true influencing factors [35], leading to extended processing times during the analysis of individual variables. This often results in issues such as multicollinearity and abnormal variance inflation factors (VIF). To address these challenges, a new set of independent variables was developed within the boundaries of a hexagonal grid. This approach averages the data values based on the size of the hexagonal area, resulting in data that is more uniform and continuous than point data. It also better reflects the actual patterns of infection, typically arising from the interconnection of water flow lines near communities, as illustrated in Fig. 6. Additionally, the independent variable dataset was classified into hexagonal units of area. The optimal grid size of 2.1 km is adjusted and organized to create an index of independent variables derived from mathematical models. Once the grid is established, it is utilized to extract values for eight independent variables, which include both vector and raster indexes.

The area of interest (AOI) was defined as the Sakon Nakhon Province, with the download period set from ‘2019-12-01’ to ‘2021-01-31’ to align the imagery with the liver leafworm data. 2) Images with cloud cover exceeding 10 % were filtered out to minimize reflection discrepancies in the calculation of the independent variable indices related to moisture and vegetation. This analysis includes various data layers assessing land use suitability for the habitats of intermediate hosts, soil properties conducive to habitation, proximity to roads, proximity to water sources, accumulated distance from the gastric water flow line, mean surface cover temperature during the dry season, and both the average and mean values of the dry season surface cover index [1,20,21]. These variables are denoted by symbols X1 to X8, as outlined in Eqs. (1), (2), (3), (4), (5), (6), (7), (8), (9). The eight equations are descriptive and representative of the symbols used in the alternative modeling, as shown in Table 2. Additionally, the equation used to calculate the size of the hexagonal grid area aims to ensure equal geographical weighting. The grid features a diagonal size of 2.1 km, determined by the average length of the river and the dimensions of the study area for the two districts, as indicated in Eq. (2).

(1) $$X_{1}=\frac{\sum_{i=1}^{n}L_{i}W_{i}}{\sum_{i=1}^{n}L_{i}A_{k}W_{i}}$$

**Table 2:** Data sources, preprocessing, and relevance to liver fluke infection (OV).

Independent Variable (X)	Full Name	Data Source	Preprocessing Steps	Temporal Alignment with Infection Data	Relevance to OV Infection
X1	Land Use	Sentinel-2 (ESA)	Selected land use types suitable for intermediate hosts (e.g., wetlands, rice paddies)	Satellite imagery from 2019 to 12-01 to 2021-01-31	Agricultural and open water areas serve as habitats for intermediate hosts such as snails and fish
X2	Soil Drainage	Land Development Department (LDD)	Converted to suitability scores for drainage types	Latest available data aligned with infection period	Well-drained soils facilitate host embedding and survival, increasing infection risk
X3	Road Network	OpenStreetMap / Department of Highways	Created buffer zones of 500–2000 m around roads	Road data from 2019 to 2021	Areas near roads often host communities and fertilizer use, which may contaminate water sources with parasite eggs
X4	Water Sources	Department of Water Resources / Sentinel-2	Created distance buffers from water bodies	Same imagery period as infection data	Water bodies are key habitats for intermediate hosts and contact points for human exposure
X5	Stream Lines	HydroSHEDS (WWF) / DEM (12.5 m)	Calculated cumulative flowline distances within hexagons	Hydrological data matched to infection timeframe	Stream networks facilitate downstream spread of parasite eggs from upstream sources
X6	Surface Temperature	MODIS (NASA) / Sentinel-2	Filtered imagery with <10 % cloud cover; calculated dry-season surface temperature averages	Dry-season imagery from 2019 to 2021	Surface temperatures below 30 °C support intermediate host survival and parasite development
X7	NDMI (Moisture Index)	Sentinel-2 (ESA)	Filtered cloud cover; calculated NDMI averages per hexagon	Dry-season imagery from 2019 to 2021	High surface moisture promotes host embedding and reproduction, increasing infection potential
X8	NDVI (Vegetation Index)	Sentinel-2 (ESA)	Filtered cloud cover; calculated NDVI averages per hexagon	Dry-season imagery from 2019 to 2021	Vegetation cover helps retain moisture and provides food sources for intermediate hosts like snails

Where X_1_ indicates the suitability of land use for habitats of intermediate hosts. This is determined by calculating the list of suitable land use types for the temporary residence of the central host. *W*_*i*_ denotes the value of a specific type of land use, where *i* corresponds to (1 = built-up, 2 = woodland, 3 = miscellaneous, 4 = paddy field, and 5 = rice paddies in irrigated areas and water body). *L*_*i*_ represents the area of land use type *i*, measured in square meters. The area of a regular hexagon can be calculated using the following formula. Where (*a*) represents the length of the sides of the hexagon, and *A*_*k*_ represents the area in any unit of measurement for polygon *k*. This is used as the divisor of space in all subsequent models. Demonstrating the calculation of the area of a hexagon as presented in Eqs. (2), (3), respectively.

(2) $$A_{k}=\frac{3\sqrt{3}}{2}\;x\;{\left(a\right)}^{2}$$

(3) $$X_{2}=\frac{\sum_{i=1}^{n}{\mathrm{Sd}}_{i}W_{i}}{\sum_{j=1}^{m}A_{k}W_{i}}$$

When $X_{2}$ indicates soil properties suitable for habitation, this is based on the observation of drainage patterns in the average soil accumulated within the hexagonal area. ${\mathrm{Sd}}_{i}$ indicates the drainage type for any soil type. $W_{i}$ is represents the drainage weight value for any type of soil. As shown in Eq. (4).

(4) $$X_{3}=\frac{\sum_{i=1}^{n}L_{i}}{A_{k}}$$

Where $X_{3}$ represents the total buffer area distance corresponding to the optimal distance ranges for the road network within a hexagonal grid. $L_{i}$ represents the buffer distance for any phase i, ranging from 500 m to over 2000 m. The buffer distance is determined by calculating the average distance between agricultural plots located near the road and those situated more than 2 km away. This distance range is then divided into intervals of 500 m to enhance visibility on the map. It is standardized within a scale of 0 to 1 to allow for thorough measurement of values. As shown in Eq. (5).

(5) $$X_{4}=\frac{\sum_{i=1}^{n}\sum_{j=1}^{m}L_{i}A_{j}}{A_{k}}$$

Where $X_{4}$ represents the total distance of the multipliers between the buffer phases at any given *i*-distance from the water bodies located within the hexagonal area. $L_{i}$ is the same distance from the boundary as shown earlier. $A_{j}$ represents the area of the neighboring boundary at a distance of *j*, measured in square meters. As shown in Eq. (6).

(6) $$X_{5}=\frac{\sum_{i=1}^{n}\sum_{j=1}^{m}{\mathrm{DS}}_{i}A_{j}}{A_{k}}$$

Where $X_{5}$ is the sum of the products of the accumulated distances measured by the streamlines distributed within the hexagon, along with the area covered by those distances. ${\mathrm{DS}}_{i}$ represents the total length of any *i* flowline within a hexagonal grid. As shown in Eq. (7).

(7) $$X_{6}=\frac{\sum_{i=1}^{n}{\mathrm{ST}}_{i}A_{\mathrm{ik}}}{A_{k}}$$

When $X_{6}$ represents the average temperature of the surface area covered during the dry season, it serves as the average within the hexagonal area used to evaluate the suitability of the soil surface for embedding the intermediate host, which prefers conditions within a temperature range not exceeding 30 degrees Celsius [1]. ${\mathrm{ST}}_{i}T_{i}$ represents any surface temperature in degrees Celsius on the hexagonal grid. $A_{\mathrm{ik}}$ represents the overall temperature within the hexagonal boundary, measured at *k* degrees Celsius. As shown in Eq. (8).

(8) $$X_{7}=\frac{\sum_{i=1}^{n}{\mathrm{NDMI}}_{i}A_{\mathrm{ik}}}{A_{k}}$$

In this context, $X_{7}$ represents the average surface moisture during the dry season, derived from the hexagonal grid average surface moisture index. This index is utilized to evaluate the suitability of the host media for subsurface embedding. The percentage of soil surface moisture is adjusted to fall within the standard range of 0–1, with the average dry season exhibiting an optimal range of 0.4–0.7 [1]. ${\mathrm{NDMI}}_{i}$ denotes the value of surface wetness at any given grid location. The total surface moisture area at *i*, contained within the sub-basin boundary at *k*, is denoted by $A_{\mathrm{ik}}$. As shown in Eq. (9).

(9) $$X_{8}=\frac{\sum_{i=1}^{n}{\mathrm{NDVI}}_{i}A_{\mathrm{ik}}}{A_{k}}$$

Where $X_{8}$ represents the average percentage of vegetation cover during the dry season, defined as the average vegetation index of any hexagonal grid. This index is utilized to evaluate the suitability of the medium host for subsurface embedding within that sub-basin. ${\mathrm{NDVI}}_{i}$ can denote any value for the grid-normalized differential vegetation index. Within the sub-basin boundary at *k*, $A_{\mathrm{ik}}$ indicates the total area of the vegetation index at *i*.

### 2.4 Alternative geographically weighted regression (GWR) models

The Geographically Weighted Regression (GWR) model used in this study involves several key steps. First, vector grid data is prepared for the hexagons, which represent variables based on the percentage of infected individuals. Next, all independent variables are analyzed to create an alternative model that includes only those variables exhibiting similar correlation values, as determined from the dendrogram. This analysis leads to the grouping of the alternative model into three distinct models, represented as functions in Eqs. (10), (11), (12).

(10) $$\;{\mathrm{Model}}_{1}\left(Y\right)=\beta_{0}\left(u_{i}v_{i}\right)+\beta_{2}\left(u_{i}v_{i}\right)x_{2}+\beta_{3}\left(u_{i}v_{i}\right)x_{3}$$

(11) $$\;{\mathrm{Model}}_{2}\left(Y\right)=\beta_{0}\left(u_{i}v_{i}\right)+\beta_{4}\left(u_{i}v_{i}\right)x_{4}+\beta_{5}\left(u_{i}v_{i}\right)x_{5}+\beta_{7}\left(u_{i}v_{i}\right)x_{7}++\beta_{8}\left(u_{i}v_{i}\right)x_{8}$$

(12) $$\;{\mathrm{Model}}_{3}\left(Y\right)=\beta_{0}\left(u_{i}v_{i}\right)+\beta_{1}\left(u_{i}v_{i}\right)x_{1}+\beta_{6}\left(u_{i}v_{i}\right)x_{6}$$

In this context, $\left(u_{i}v_{i}\right)$ represents the orthogonal coordinates at each linear regression point, while $\beta_{k}\left(u_{i}v_{i}\right)$ denotes the estimated regression coefficient at those points. Furthermore, the regression coefficient $\left(\beta \right)$ for each independent variable (*X*) is estimated as a matrix with dimensions n × (k + 1) at each linear regression point [36]. The GWR model analyzes multiple linear regression equations at each regression point [37], applying weights to emphasize the data [[38], [39], [40], [41], [42]]. The regression coefficient is subsequently estimated, as illustrated in (Eq. (13)).

(13) $$\beta_{\left(i\right)}={\left(X^{T}W\left(i\right)X\right)}^{-1}X^{T}W\left(i\right)y$$

The next step in the process involves selecting the kernel function, which is critical for assigning weights to nearby data points. The Gaussian kernel has been chosen for its appropriateness in handling data represented in uniformly distributed spatial units [[42], [43], [44]], such as a hexagonal grid. The fixed bandwidth method is set with a maximum distance of 2100 m, ensuring it does not exceed twice the size of the hexagonal grid, which is limited to 1050 m. Subsequently, the model calculates the weight for each data point using the specified kernel function and bandwidth.

In the GWR modeling procedure, each data point is analyzed using a regression model based on approximate data. The calculated weights serve as the regression coefficients for each independent variable at each data point [45]. Each model's performance is assessed using R-squared values, which indicate the model's suitability, while the RMSE value evaluates the expected percentage of infections. The results are then analyzed to interpret the coefficients obtained for each area, helping to understand the relationships between the variables. These coefficients are subsequently used to create a map for spatial analysis.

To evaluate the accuracy of the GWR, we employed the Receiver Operating Characteristic-Area Under Curve (ROC-AUC) technique. This method is widely used in machine learning to assess the performance of different models and to identify potential issues related to interpretation and criteria selection [1].

The evaluation of the predictive performance of the percentage of OV infections in hexagonal units employs Receiver Operating Characteristic (ROC) analysis and Area Under the Curve (AUC) metrics to assess the effectiveness of the classification model [46]. The binary infection status is defined as infection (1) or no infection (0), with predicted values representing the probability or score generated by the model [47]. The ROC curve is utilized to determine the relationship between the True Positive Rate (TPR) the proportion of actual positives correctly identified and the False Positive Rate (FPR) the proportion of actual negatives incorrectly identified as positives. TPR and FPR are calculated by varying the probability threshold from 0 to 1, leading to the computation of the AUC, which represents the area under the ROC curve.

An AUC of 1 indicates a perfect model, while an AUC of 0.5 suggests that the model's performance is equivalent to random guessing [48]. An AUC greater than 0.7 signifies good performance, and an AUC greater than 0.9 indicates very good performance. Finally, the spatial results are presented in map format, illustrating the percentage of infections predicted by the model. Appropriate thresholds should be established to achieve a high true positive rate (TPR) and a low false positive rate (FPR). The spatial results are then compared with actual data to further validate the model's accuracy [49].

## 3 Results

### 3.1 Spatial patterns of variables

**Fig. 7:**
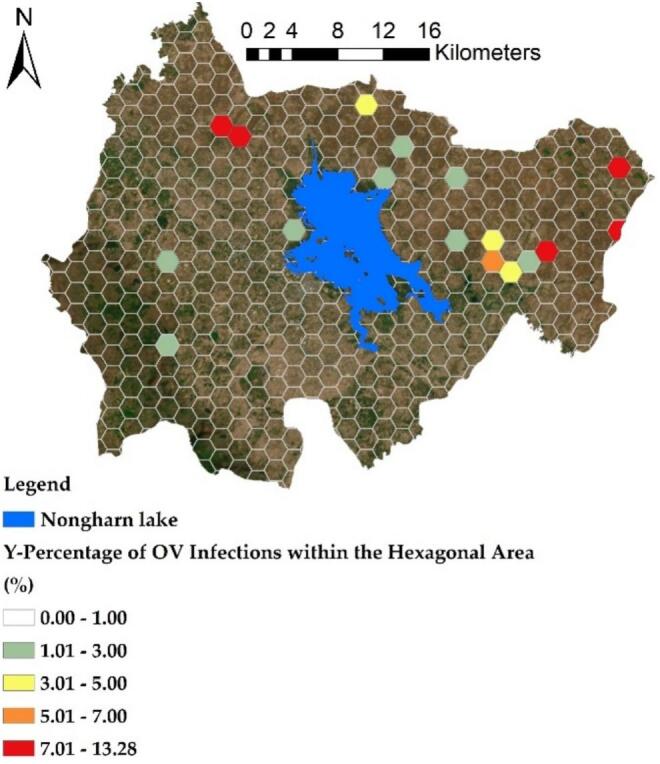
The hexagonal grid of prevalence of ov-infection in terms of percentage Y (prevalence of OV, %).

The distribution model for the dependent and independent variables was organized into a hexagonal grid with a diagonal distance of 2100 m. This grid was based on the average density of waterline length in the study area of the two districts, as previously discussed. Fig. 7 illustrates the results of this variable data generation, which is based on the prevalence of infection in water sources. The data is presented as points in a hexagonal format indicating infection percentages, with ranges divided into increments of 1 % for greater detail. The areas with the highest infection prevalence are represented in red, indicating infection percentages from 7 % to 13.28 % across 5 grids. Conversely, the range of 1 to 3 % shows the largest distribution, encompassing 7 grids. The size of the grid, being larger than the individual points, serves to encompass multiple points representing water source infections. This approach calculates an average to better represent the grid areas, thereby reducing variability in infection values from nearby water sources and enhancing the connectivity of the water sources through the integrated flow of the waterline.

**Fig. 8:**
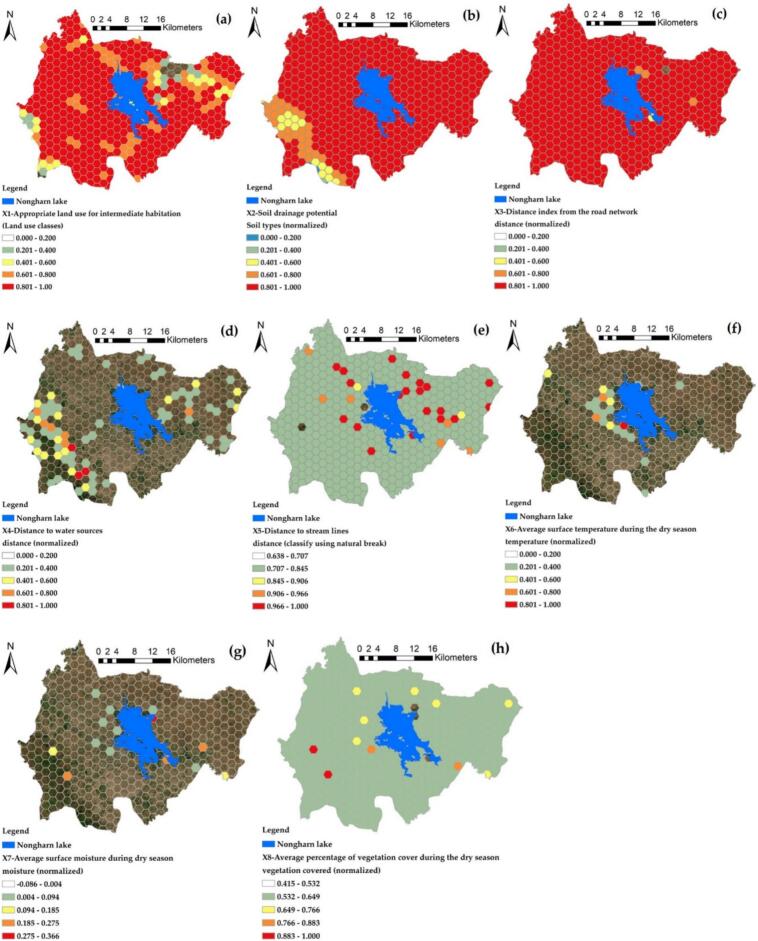
The hexagonal grid model of independent variables X1 to X8 (labeled (a) to (h)) is derived using a mathematical index map.)).

The distribution of independent variables within the hexagonal grid is determined using a mathematical model that enhances the relationship of each variable to a hexagon, enabling fair calculation of representative values. Variable X1 is illustrated in Fig. 8(a). The majority of grid values exceed 62 %, indicating the suitability of agricultural land use near water sources where moisture persists during the dry season. Most of these values are scattered throughout the entire study area, represented as a red grid, based on normalized values that can be compared with other variables. Variable X2 is illustrated in Fig. 8(b), where most grid values exceed 88 %. This indicates that the soil types are suitable for retaining moisture during the dry season due to their good drainage properties. In contrast, poor drainage characterizes the soil in most areas of the study, represented by a red grid. The results from the hexagonal grid surrounding the road network, up to a distance of 1000 m and represented by the red grid in Fig. 8(c), indicate that the road network is evenly distributed across the study area. This distribution plays a significant role in retaining water that overflows from the Mekong River to the Nong Harn Lake, leading to the formation of small water sources along the intersecting roads during the dry season. In contrast to the X4 factor effect, which indicates proximity to freshwater bodies in low-fidelity areas, the highest number of grids falls within the ranges of 0.000 to 0.200 (51 %) and 0.201 to 0.400 (22 %). Since the buffer distance scoring range extends no more than 750 m to ensure suitable habitat for the medium, most grids are classified as low suitability. This necessitates a screening of grids located very close to the water source, as they could serve as models for future sample collection of infected fish, as illustrated in Fig. 8(d). The final proximity factor is determined by the flow line of gastric water, which is likely a crucial element for the transfer of habitat and the connection between the gastric water source and the Nong Harn Swamp. The representation of the suitable range was adapted using the natural breaks method to accurately reflect the actual range values based on the number of sample grids. This analysis revealed that the highest values were found in the grid surrounding the Nong Harn Lake, with a gradual decrease in values as the distance from the swamp increased. This trend indicates a decline in humidity as one moves away from the main moisture source, represented as the X5 variable, as illustrated in Fig. 8(e). During the dry season, the average surface temperature analysis revealed that most areas did not exhibit high-temperature or red grids. Instead, a moderate temperature range of 25–32 degrees Celsius was observed, particularly near the Nong Harn Lake, which is primarily a community area. This environment allows buildings to retain soil surface moisture effectively during the dry season. The impact of the X6 variable is illustrated in Fig. 8(f).

**Fig. 9:**
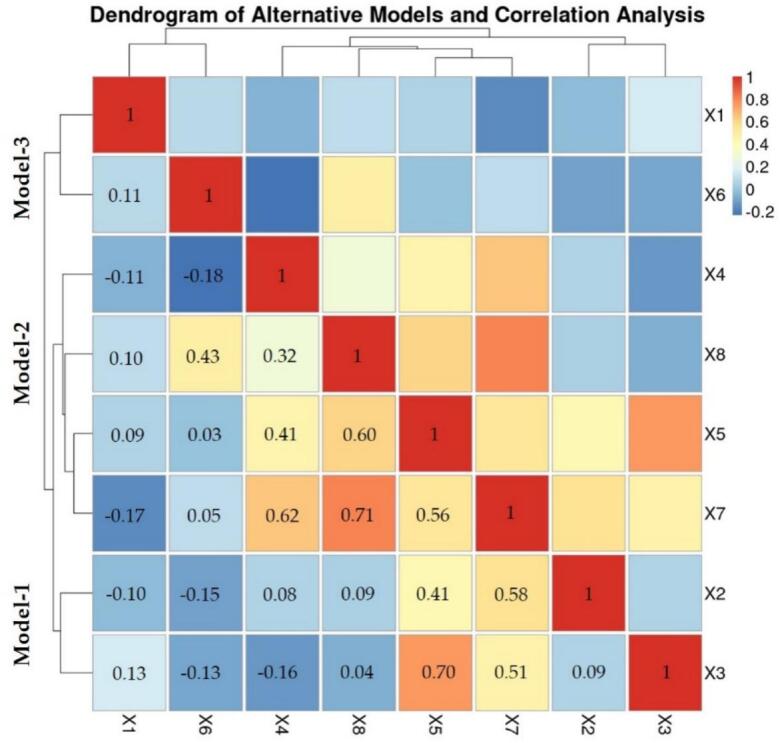
The correlation matrix of independent variables used for grouping alternative models.

The results of the soil surface moisture index, represented as X7, are illustrated in Fig. 8(g). This figure indicates that 97 % of the grid area possesses suitable humidity levels for habitat, with values ranging from −0.086 to 0.094. This index specifically reflects water or moisture at a depth of no more than 15 cm beneath the soil surface. Additionally, Fig. 8(h) presents the vegetation surface cover, which shows a consistent relationship with the X7 variable. Furthermore, Fig. 9 reveals a correlation value of 0.71. These findings demonstrate a mutual support between vegetation cover and soil surface moisture retention; as plant cover increases, the ability to retain moisture in the soil also improves. The eight independent variables were screened and organized into alternative models to enhance the use of data and spatial models.

Importing all eight independent variables to create the GWR model may not accurately represent the data, as this could lead to a high VIF value. Therefore, the factors analyzed through correlation and screened using VIF values should be reduced to no more than four [50]. These can be summarized into three groups, as illustrated in Fig. 6. The first group includes soil properties favorable for intermediate host habitats and proximity to the road network. Model-2 consists of four independent variables: proximity to freshwater sources, proximity to gastric flow lines, vegetation soil cover, and soil surface moisture indices, represented by NDVI and NDMI. Model-3 includes two independent variable sets: land use that supports intermediate host habitats and the average soil surface temperature during the dry season.

### 3.2 Spatial modeling for prediction of liver fluke (*Opisthorchis viverrine*, OV) infection

**Fig. 10:**
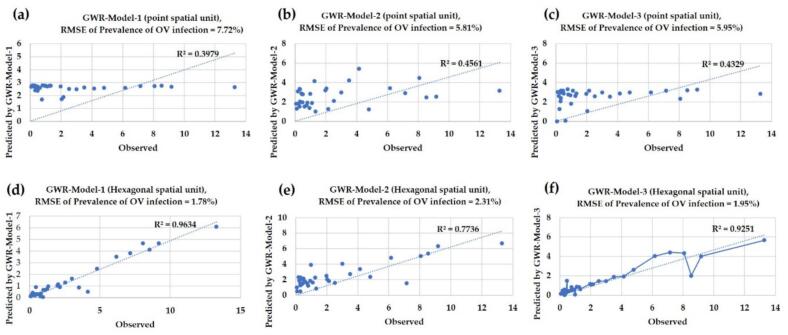
Comparison of the RMSE for (a) Model-1, (b) Model-2, and (c) Model-3.

The results of the water source infection prediction model present the statistical values and performance metrics for each model, as illustrated in Table 3. The table provides a comparison of the GWR models, specifically Model-1, Model-2, and Model-3, in both point data and hexagonal grid modeling formats. In Model-1, the t-statistic was employed to determine whether the coefficients in each area differed statistically significantly from zero. This was achieved by comparing the critical values from the t-statistic and *p*-value distributions of the X2 and X3 variables. The t-statistic of 3.021 exceeded the critical value of +/− 2.042, and the p-value of 0.032 was significantly lower than the significance level of 0.05. These results indicate that the coefficients of the independent variables are significant. This finding is consistent with Model-3, where the X1 variable had a t-statistic of 2.045 and a p-value of 0.022, while the X6 variable had a t-statistic of −2.453 and a p-value of 0.045. In contrast, Model-2 did not support the acceptance of any of the four variables. The performance metrics for Models 1, 2, and 3 showed increases of 58.7 %, 41.1 %, and 53.2 %, respectively. However, the prediction error for these models also increased to 77.12, 60.2 %, and 67.2 %, respectively. Additionally, the accuracy results are illustrated in the graph presented in Fig. 10.

**Table 3:** Results of the GWR for the chosen alternative model.

GWR-models	Factors	Coefficients	t-stat	p-value	R^2^(GWR) point	R^2^(GWR) hexagonal	R^2^ difference	RMSE (%) difference
Model-1	Intercept	12.431	1.458⁎⁎⁎	0.000 ⁎⁎⁎	0.397	0.963	+58.77 %	+77.12
	X2 (soil drainage)	6.048	3.021 ⁎⁎⁎	0.032⁎⁎⁎				
	X3 (distance to road)	−5.185	−2.027 ⁎⁎⁎	0.025 ⁎⁎⁎				
Model-2	Intercept	72.452	3.184 ⁎⁎⁎	0.000 ⁎⁎⁎	0.456	0.773	+41.01 %	+60.24
	X4 (distance to water sources)	−5.182	−2.093 n/s	0.052 n/s				
	X5 (distance to stream lines)	−4.365	1.867 n/s	0.061 n/s				
	X7 (NDMI)	−8.962	−2.846 n/s	0.238 n/s				
	X8 (NDVI)	−9.795	−2.749 n/s	0.375 n/s				
Model-3	Intercept	8.467	2.749⁎⁎⁎	0.000 ⁎⁎⁎	0.432	0.925	+53.29 %	+67.22
	X1(land use)	5.167	2.045 ⁎⁎⁎	0.022 ⁎⁎⁎				
	X6 (surface temperature)	−1.264	−2.453 ⁎⁎⁎	0.045 ⁎⁎⁎				

⁎⁎⁎ = significant at 5 % level. n/s = not significant. Results of Monte Carlo test for spatial non-stationarity.

The results presented in Fig. 10(a) to (c) indicate that the GWR model built from point data predicts values that closely align with actual measurements, as reflected in the R^2^ values, which range from 0.397 to 0.456. This model utilizes the same set of independent variables as the hexagonal grid model. Among the different models, Model-1 exhibited the lowest R^2^ value, followed by Model-3, while Model-2 achieved the highest R^2^. The addition of a large set of independent variables in point space contributed to the improved R^2^ values. Additionally, the RMSE values aligned with the R^2^ findings, demonstrating a tolerance range of 5.81 % to 7.72 %. The slope of the graph indicated a more effective relationship, with independent variables demonstrating a better ability to predict infections and a lower tolerance. The RMSE for the point model was consistently higher than in every test for the other models, ranging from 1.78 % to 2.31 %.

In contrast to the residual map of the GWR model created using hexagonal grid data, the standard deviation (SD) values reflect the results of Model-1, Model-2, and Model-3, respectively. The red grid indicates areas where the model's predicted values exceed the actual values, while the blue grid highlights areas where the model predicts an infection level lower than what actually occurs. Therefore, the application of the model's results should take into account not only the significant independent variable factors but also the effect of prediction deviation [51].

**Fig. 11:**
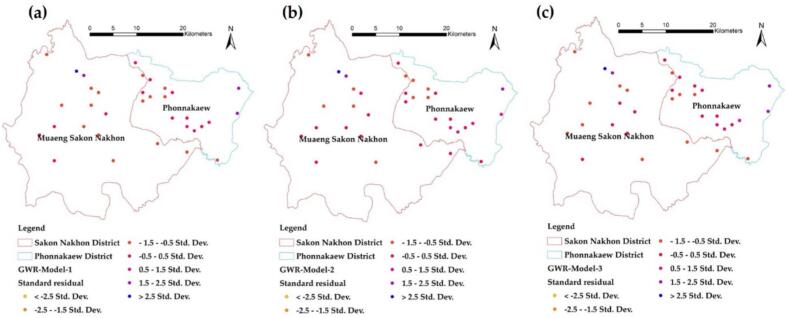
Comparison of standard residuals from alternative GWR models at points: (a) Model-1, (b) Model-2, and (c) Model-3.

**Fig. 12:**
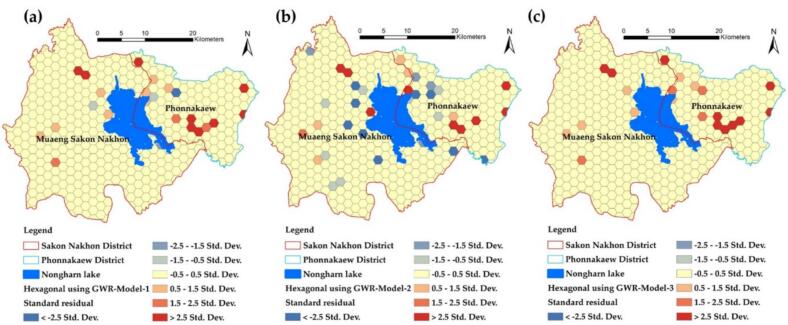
Comparison of standard residuals from different GWR models at hexagonal grids: (a) Model-1, (b) Model-2, and (c) Model-3.

In addition to presenting the predictions in a tabular format, this study includes a residual map of the GWR model generated from point data, as illustrated in Fig. 11(a), (b), and 11(c). The results indicated that some predicted values were higher than the actual values, while others were lower than those close to the actual infection values. This discrepancy is attributed to the low R^2^ values and high RMSE values observed across all models. In contrast, the residual map of the GWR model based on hexagonal grid data reveals the standard deviation (SD) of the results from Model-1, Model-2, and Model-3, as shown in Fig. 12(a), (b), and (c). Analysis revealed that over 87 % of the grids across all models had SD values within a low and acceptable range, specifically between −0.5 and 0.5, represented in light yellow. Grids colored in red indicate predictions where the model overestimates values compared to the actual figures, while blue-highlighted grids represent instances where the model underestimates infection values. Therefore, when applying the model's results, it is essential to consider not only the significant independent variable factors but also the impact of prediction deviations.

### 3.3 Severity of the predicted results

**Fig. 13:**
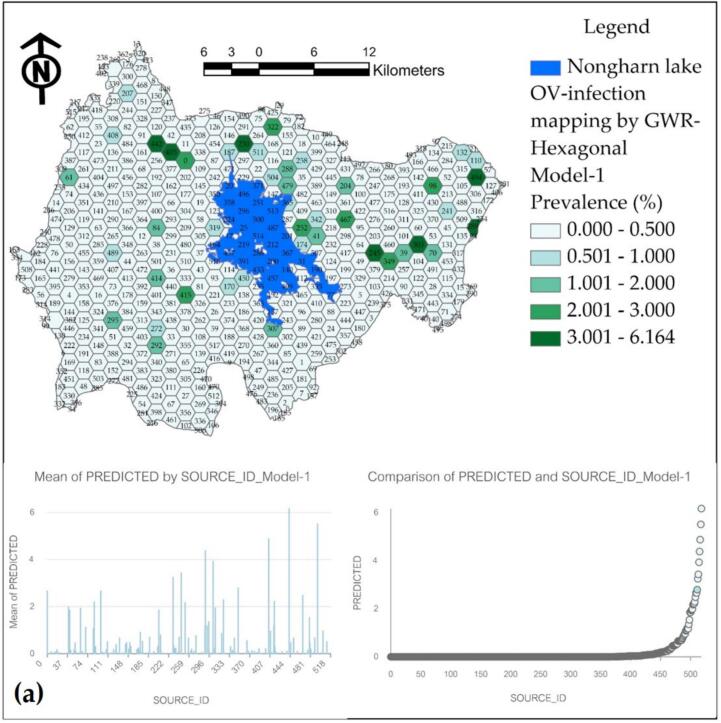

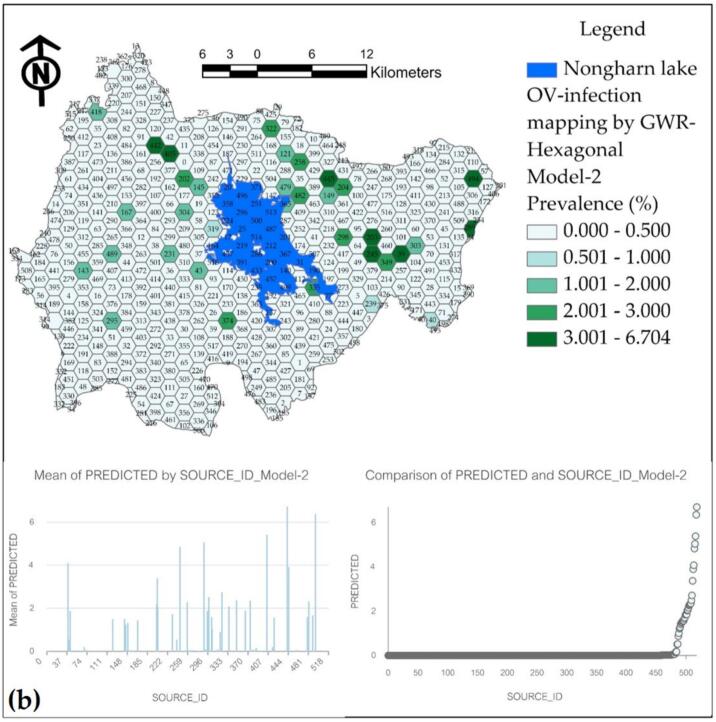

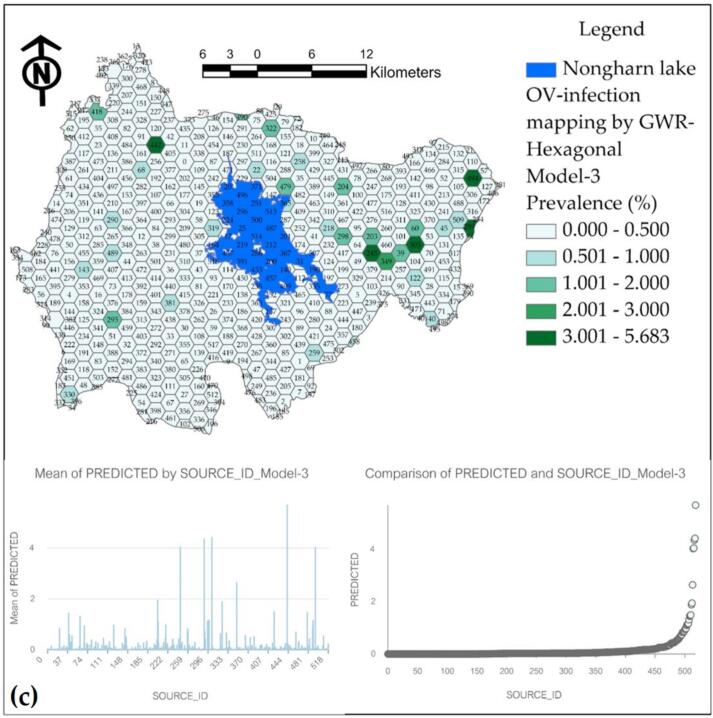
Prediction maps of OV infection prevalence from various GWR models at hexagonal grids: (a) Model-1, (b) Model-2, and (c) Model-3.

The final results of the applied GWR model are presented as a percentage of the prevalence of water source infections on a hexagonal grid, as illustrated in Fig. 13. Model-1, which exhibits the highest R^2^ value of 0.964, predicts prevalence values that do not exceed the actual survey values; however, it also diverges from the predicted results. Model-2 has been observed to produce inflated predictions across various water sources. Model-3, while having a respectable R^2^ value of 0.925, demonstrates a significant percentage of prediction discontinuity, rendering it less representative compared to Model-1.

The prediction of infection prevalence for each model is adjusted to a mean in a hexagonal format, ensuring that the maximum value reaches 3 % or more, thereby facilitating appropriate comparisons across each hexagonal grid. Fig. 13(a) illustrates that the hexagonal grid predicted a high infection prevalence percentage in 5 grids. When compared to the locations of actual high-infection water sources, it was found that there were matching locations, also totaling 5 water sources. In contrast, Model-2 predicted a maximum infection prevalence in 7 grids, while Model-3 predicted in 4 grids. This analysis confirms that Model-1 is the most effective for predictions when comparing both point and hexagonal models.

## 4 Discussion

### 4.1 Applying the model to predictive applications

**Fig. 14:**
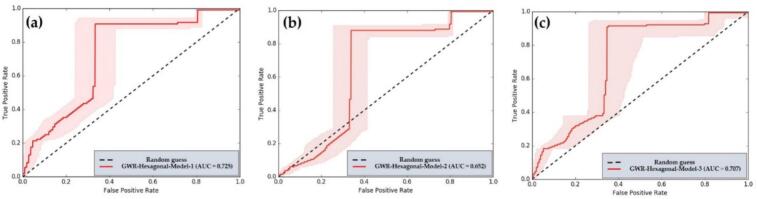
Comparison of the AUC values for (a) predictions made by Model-1, (b) predictions made by Model-2, and (c) predictions made by Model-3.

The evaluation of the predictive performance for the percentage of OV infections in hexagonal units employs the ROC curve and the AUC to assess the effectiveness of the classification model. In this binary classification, infections are categorized as either infected (1) or not infected (0), with predicted values representing the probabilities or scores generated by the model. The ROC curve illustrates the relationship between the True Positive Rate (TPR), which is the percentage of actual positive cases correctly identified as positive, and the False Positive Rate (FPR), which is the percentage of actual negative cases incorrectly identified as positive. TPR and FPR are calculated by varying the probability threshold from 0 to 1. The AUC, representing the area under the ROC curve, is interpreted on a scale from 0 to 1. An AUC of 1 indicates a perfect model. An AUC greater than 0.7 indicates good performance, while an AUC greater than 0.9 signifies very good performance. The validation of the three alternative models using the AUC technique revealed that Model-1 achieved the highest AUC value of 0.725, followed by Model-3 at 0.707 and Model-2 at 0.652. Furthermore, a set of independent variables can be utilized to create a spatial model for predicting infections in similar watersheds. This hexagonal model should incorporate four key independent variables: land use, soil drainage properties, proximity to the road network, and average surface temperature. The AUC model validation value is illustrated in the graph depicted in Fig. 14.

### 4.2 Tolerances of GWR models

**Fig. 15:**
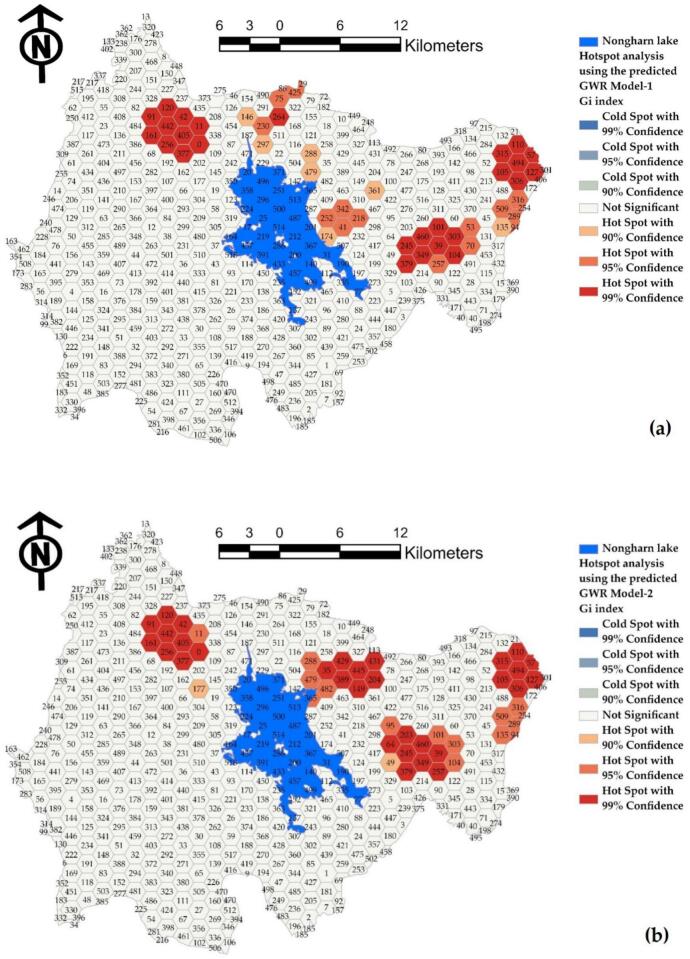

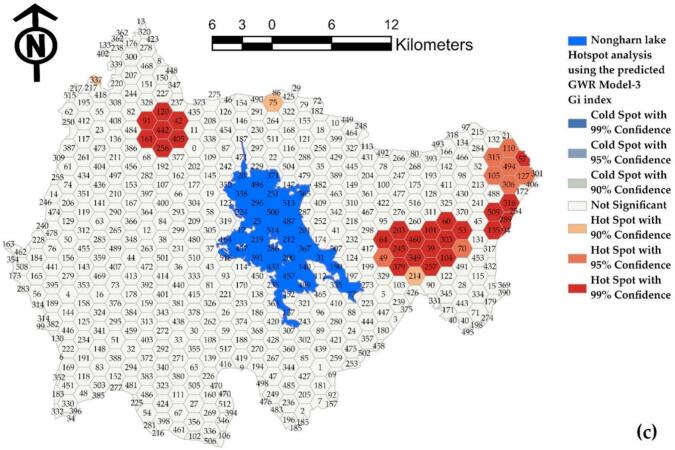
Comparison of hot spot analysis for (a) predictions from Model-1, (b) predictions from Model-2, and (c) predictions from Model-3.

The validity of water source infection predictions should be substantiated through rigorous analysis. As illustrated in Fig. 15, panels (a), (b), and (c) present the analytical outcomes for Model-1, Model-2, and Model-3, respectively. The results indicate that the predictions generated by incorporating a set of independent variables from the alternative model exhibit exaggerated values, as evidenced by the areas highlighted in red. Conversely, the predictions falling below actual infection rates are represented in blue. In many instances, the model predicts a higher level of contagion than observed due to the influence of independent variables resulting from grid averaging. Nonetheless, the model maintains an acceptable prediction accuracy exceeding 72 %, which is deemed satisfactory for spatial outbreak assessments.

The reaffirmation of the clustering of predictions through automatic spatial correlation indicates that the infection predictions were significantly clustered, with a confidence level exceeding 95 %, as illustrated in Table 4. In these figures, the results of Model-1 and Model-2, derived from clustering points, appear random, while Model-3 demonstrates clustering due to yielding similar predictions. The hexagonal grid analysis results provide clustered cohesion values for all models. The hexagonal approach not only offers a higher R^2^ value compared to point values and a better RMSE but also facilitates an easily interpretable forecast and illustrates the connectivity of the watershed by demonstrating the coherence of the forecast results.

**Table 4:** Predicted spatial autocorrelation index: points versus hexagonal representation.

Models	Moran's Index	z-score	p-value	spatial distribution characteristics
Model-1(Points)	−0.040541	−0.103977	0.917187	Random
Model-2(Points)	0.122636	1.443958	0.148751	Random
Model-3(Points)	0.224817	2.534218	0.011270	Clustered
Model-1(Hexagonal)	0.082422	3.123849	0.001785	Clustered
Model-2(Hexagonal)	0.083775	3.181694	0.001464	Clustered
Model-3(Hexagonal)	0.050403	1.986825	0.046942	Clustered

### 4.3 Advantages of adding spatial weighting within a hexagonal grid to independent variables

Developing a raster dataset of independent variables in the form of a hexagonal grid can enhance the benefits of increased spatial weights, thereby improving the accuracy of the GWR model compared to traditional spatial units, such as points. By integrating a hexagonal grid model into the GWR framework, we can achieve more precise modeling outcomes.

#### 4.3.1 Data complexity

The point-based data regarding the locations of infected water sources can be highly complex due to the extensive number of data points. This complexity complicates analysis and processing, and overlapping locations may not yield significant spatial distinctions once the narrative data is integrated. The data for independent variables is generated and stored in various forms, including linear data representing waterways and roads, polygon data for land use and soil drainage, and raster data from Sentinel-2, which comes in different ground resolutions. To effectively integrate and weigh these different data types, it is essential to use a consistent spatial unit of measurement within this hexagonal grid. The hexagonal model streamlines this complexity by partitioning the space into hexagonal cells. It enhances data structure and facilitates manipulation by employing the average of a set of independent variables as the center of each grid cell.

#### 4.3.2 Consistent spatial data visualization

Point-based data may exhibit uneven distribution, leading to inconsistencies in gaps or densities resulting from the spatial arrangement of certain variables, such as the locations of contaminated water sources. The hexagonal model features a uniform structure. This study enhances data consistency by employing grid regularities to compute averages, including NDVI, NDMI, and surface temperature satellite indices.

#### 4.3.3 Enhance effective spatial analysis

Point-based data can complicate spatial analysis, including density calculations and the identification of unique areas. The hexagonal model streamlines spatial analysis by ensuring that each cell is uniform in area and shape. Visualizing the predicted infections on a hexagonal grid pattern facilitates a clearer understanding of the connections within the watershed compared to the group-based dotted model, effectively illustrating the density of the watershed.

#### 4.3.4 Reducing edge effects

Point-based data: Edge effects may arise when data points are situated near the periphery of the area, leading to potential inaccuracies in the analysis. Hexagonal models mitigate these issues, as hexagonal structures maintain equal distances from the center to the edges in all directions. This characteristic facilitates a more effective extraction of vector molecules of independent variables compared to point data, such as road line variables and river proximity.

#### 4.3.5 Efficient processing

Point-based data processing can be time-consuming due to the extensive number of data points and potential overlaps, which may obscure spatial differences. Hexagonal models enhance processing efficiency by organizing data in a grid format.

#### 4.3.6 Reduction of statistical discrepancies

Point-based data can lead to statistical discrepancies due to inconsistencies within the data. Hexagonal models mitigate these discrepancies by organizing the data in a uniform grid pattern.

#### 4.3.7 Neighborhood analysis support

Point-based data can complicate neighborhood analysis due to the necessity of calculating distances between points. The hexagonal model streamlines neighborhood analysis, as each cell has well-defined neighboring cells.

#### 4.3.8 Applications in various aspects of the display of GWR models

Point data may be unsuitable for certain types of analyses, including density analysis or hotspot and clustering mapping. Hexagonal models are particularly well-suited for analyzing aspects such as hotspot mapping, density analysis, and spatial prediction.

In conclusion, hexagonal GWR models provide several advantages in the manipulation and analysis of spatial data compared to point-based data. However, the selection of an appropriate grid width is critical for ensuring accuracy. If the grid size is too large, the grid average may not accurately reflect the data, as the maximum and minimum values within the grid may not be evenly distributed. Conversely, if the hexagonal grid is too small, it may result in intermittent calculations of grid regularity; a greater number of smaller grids may not effectively represent the averaging of vector data, such as flow lines or road networks. Therefore, the design of an optimal grid size is essential for maintaining the accuracy of the model. The most significant advantages of developing a model on a hexagonal grid in this study include improved model accuracy: The utilization of hexagonal grids enhances the accuracy of the GWR model, achieving R^2^ values of 58.7 %, 41.1 %, and 53.2 % for Model-1, Model-2, and Model-3, respectively. Furthermore, this approach significantly reduces the tolerance of the RMSE.

Forwarding the results of the study to other areas can be guided by the guidelines established by the Sakon Nakhon Provincial Public Health Office, which has set forth protocols for the prevention and control of liver parasites and bile duct cancer. These measures are integrated into the trend map of infection prevalence, which will be utilized in campaigns aimed at eliminating parasites from fish at the sub-basin level and form a central policy of the organization. This spatial modeling approach supports sanitation management strategies and the Mekong River flow network, with the objective of breaking the cycle of parasites, as illustrated by the map of the independent set of variables.

## 5 Conclusion

The development of a spatial model to predict liver leafworm infection in a major freshwater source connected to the Mekong River is based on the Geographically Weighted Regression (GWR) model. The first step involves creating a mathematical model to adjust the stored geographic data values of various independent variables, measured by calculating the average within hexagonal grid units. In the second step, these measurements are weighted. Performance evaluations have confirmed that the hexagonal grid model enhances effectiveness, as indicated by improvements in R^2^, RMSE, and AUC metrics. However, applying this model requires testing to determine the appropriate size of the hexagonal grid for the study area. Additionally, it is crucial to gather information that connects infection characteristics in the area, such as water flow lines and road networks. This information will help establish appropriate weighting for the hexagonal grids and facilitate the creation of effective trend charts. This study serves as a prototype, with hopes for further development using machine learning models and a broader range of independent variables. This approach aims to demonstrate that modeling within hexagonal spatial units offers advantages over traditional units like points or polygons.

## CRediT authorship contribution statement

**Benjamabhorn Pumhirunroj:** Methodology, Investigation, Funding acquisition, Conceptualization. **Patiwat Littidej:** Writing – review & editing, Writing – original draft, Project administration, Methodology, Formal analysis, Data curation, Conceptualization. **Thidarut Boonmars:** Writing – review & editing, Validation, Supervision, Resources, Data curation. **Atchara Artchayasawat:** Validation, Supervision, Resources. **Nutchanat Buasri:** Visualization, Validation. **Donald Slack:** Writing – review & editing, Supervision.

## Declaration of competing interest

The authors declare that they have no known competing financial interests or personal relationships that could have appeared to influence the work reported in this paper.

## Data Availability

No data was used for the research described in the article.
